# Supplementary material for: A fault-intrusion-tolerant system and deadline-aware algorithm for scheduling scientific workflow in the cloud
Source: PeerJ Comput Sci. 2021 Nov 2;7:e747. doi: 10.7717/peerj-cs.747 (PMC8576554; doi:10.7717/peerj-cs.747)
Supplement: Supplemental Information 6 [file peerj-cs-07-747-s006.zip › Myproject1.1/docs/org/workflowsim/utils/class-use/ClusteringParameters.ClusteringMethod.html]

Uses of Class org.workflowsim.utils.ClusteringParameters.ClusteringMethod


JavaScript is disabled on your browser.


- Overview
- Package
- Class
- Use
- Tree
- Deprecated
- Index
- Help

- Prev
- Next

- Frames
- No Frames

- All Classes

## Uses of Class org.workflowsim.utils.ClusteringParameters.ClusteringMethod

- Packages that use ClusteringParameters.ClusteringMethod

  | Package | Description |
  | --- | --- |
  |  |  |
  | --- | --- |
  | org.workflowsim.utils |  |
- - ### Uses of ClusteringParameters.ClusteringMethod in org.workflowsim.utils

    Methods in org.workflowsim.utils that return ClusteringParameters.ClusteringMethod

    | Modifier and Type | Method and Description |
    | --- | --- |
    |  |  |
    | --- | --- |
    | `ClusteringParameters.ClusteringMethod` | ClusteringParameters.`getClusteringMethod()` Gets the clustering method |
    | `static ClusteringParameters.ClusteringMethod` | ClusteringParameters.ClusteringMethod.`valueOf(java.lang.String name)` Returns the enum constant of this type with the specified name. |
    | `static ClusteringParameters.ClusteringMethod[]` | ClusteringParameters.ClusteringMethod.`values()` Returns an array containing the constants of this enum type, in the order they are declared. |

    Constructors in org.workflowsim.utils with parameters of type ClusteringParameters.ClusteringMethod

    Constructor and Description

    `ClusteringParameters(int cNum,
    int cSize,
    ClusteringParameters.ClusteringMethod method,
    java.lang.String code)`

    Initialize a ClusteringParameters

- Overview
- Package
- Class
- Use
- Tree
- Deprecated
- Index
- Help

- Prev
- Next

- Frames
- No Frames

- All Classes
